# Low circulating serum levels of second mitochondria-derived activator of caspase (Smac/DIABLO) in patients with bladder cancer

**DOI:** 10.3892/ijo.2012.1324

**Published:** 2012-01-03

**Authors:** YOICHI MIZUTANI, YOJI KATSUOKA, BENJAMIN BONAVIDA

**Affiliations:** 1 Department of Urology, Faculty of Medicine, Osaka Medical College, Osaka 569-8686, Japan; 2 Department of Microbiology, Immunology and Molecular Genetics, Jonsson Comprehensive Cancer Center, UCLA School of Medicine, University of California at Los Angeles, CA 90095, USA

**Keywords:** Smac/DIABLO, bladder cancer, prognosis

## Abstract

Smac/DIABLO promotes apoptosis by antagonizing inhibitor of apoptosis proteins. The expression of Smac/DIABLO in tissues has been reported in various cancers; however, little is known about circulating levels of Smac/DIABLO. The present study was designed to first determine if Smac/DIABLO can be detected in the serum and then assess whether the circulating levels of Smac/DIABLO are of prognostic significance in patients with bladder cancer. The levels of Smac/DIABLO in the sera of 173 patients with bladder cancer and 36 normal donors were determined by using an enzyme-linked immunosorbent assay. The mean serum level of Smac/DIABLO in patients with bladder cancer was approximately 2-fold lower than that in normal donors. The mean level of serum Smac/DIABLO in patients with muscle-invasive bladder cancer was lower than that in patients with non-muscle invasive cancer. In addition, the mean serum Smac/DIABLO level in patients with T4 muscle-invasive bladder cancer was lower than that in patients with T2 and T3 cancers. The mean serum level of Smac/DIABLO in patients with Grade 3 bladder cancer was lower than that in patients with Grade 1 and Grade 2 cancers. Analysis by Kaplan-Meier revealed that patients with Ta and T1 non-muscle invasive bladder cancer with high level of serum Smac/DIABLO (more than mean value) had a longer postoperative tumor-free interval than those with low level (less than mean value) in the 3-year follow-up. Furthermore, patients with T2–T4 muscle-invasive bladder cancer with high serum Smac/DIABLO level (more than mean value) had a higher postoperative disease-free rate when compared with patients with low level (less than mean value) in the 5-year follow-up. The present study is the first to analyze circulating levels of Smac/DIABLO in the serum. The findings demonstrate that the mean serum level of Smac/DIABLO was downregulated in patients with bladder cancer compared to control healthy individuals, especially high grade muscle-invasive bladder cancer. Noteworthy, lower serum level of Smac/DIABLO predicted early recurrence in patients with bladder cancer. Overall, the findings suggest that measuring the levels of Smac/DIABLO in the serum may be considered a prognostic parameter in patients with bladder cancer. Furthermore, Smac/DIABLO may be a molecular therapeutic target in bladder cancer.

## Introduction

Bladder cancer accounts for ~4% of all cancers worldwide. Clinical factors have been used historically as prognostic or predictive markers such as stage and grade of bladder cancer. Recent advances have been achieved to unravel both the pathogenesis and molecular biology of bladder cancer with the objective of developing new methods for early diagnosis and better prognosis for patients with bladder cancer (1). Although many factors have been reported, a few maintained independent significance in terms of overall survival (2). In addition, several reported results have been controversial by studying the same biomarker. Therefore, it is necessary to identify new reliable diagnostic and prognostic markers in bladder cancer.

Cancer cells respond to cytotoxic therapies by activation of the type II mitochondrial apoptotic pathway. The activation of this pathway results in the depolarization of the mitochondrial membrane potential and the release from the mitochondria into the cytosol both cytochrome c and second mitochondria-derived activator of caspase/direct inhibitor of apoptosis protein (IAP)-binding protein with low pl (Smac/DIABLO). These lead to the activation of caspases 3, 8 and 9 and apoptosis (3). Smac has been identified as a protein that stimulates the activation of caspase 3 in cell extracts (4). DIABLO has been identified independently and has been shown to be the same molecule as Smac and hence, it has been term Smac/DIABLO (5). Smac/DIABLO is expressed in most adult human tissues at different levels. It is synthesized in the nucleus as a precursor protein of 239 amino acid residues and an amino terminal mitochondrial leader sequence (MLS). Upon mitochondrial import, the MLS is removed by proteolysis, exposing the IAP-binding motif (IBM) at the N-terminus of the mature Smac/DIABLO and which is present as dimers. Through this IBM, Smac/DIABLO can bind IAP family members including X-linked IAP (XIAP), cIAP-1 and cIAP-2 and survivin. One way by which Smac/DIABLO promotes the activation of caspases is by displacing caspase 9 that is coupled with XIAP (3). The inhibitory function of Smac/DIABLO relies on IBM. Thus, Smac/DIABLO functions by inhibiting the IAPs; hence, Smac/DIABLO is considered as an apoptogenic factor.

Previous studies have reported that overexpression of Smac/DIABLO sensitizes the resistant cancer cells to death receptor- or anticancer cytotoxic agent-induced apoptosis (6,7). These findings suggested that Smac/DIABLO plays an important role in the regulation of apoptotic responses in cancer cells to both immune- and drug-mediated treatments. Several studies examined the expression of Smac/DIABLO in various cancers and normal tissues (8,9). However, whether Smac/DIABLO is released into the circulation and present in the serum of normal and cancer patients has not been examined. We have reported that the expression of Smac/DIABLO in patients with bladder cancer, as examined by Western blotting, is diminished (9). Further, the low level of Smac/DIABLO expression was proposed to be a prognostic factor. The present study investigated the following: 1) Whether Smac/DIABLO is detected in the serum of normal and cancer patients. 2) Whether the serum level of Smac/DIABLO decrease in patients with bladder cancer compared to normal healthy individuals. 3) Whether the level of Smac/DIABLO correlate with both the stage and grade of bladder cancer, and 4) whether the serum level of Smac/DIABLO predict tumor recurrence.

## Materials and methods

### Patients

Peripheral blood was obtained from 173 patients with initial primary bladder cancer before surgery or anticancer therapy. They included 140 male and 33 female patients, ranging in age from 24 to 89 years. Histologic diagnosis revealed that all patients had urothelial carcinoma of bladder cancer. Their histologic classification and staging according to the TNM classification were: Tis (n=8), Ta (n=89), T1 (n=45), T2 (n=9), T3 (n=16), T4 (n=6), and G1 (n=51), G2 (n=63), G3 (n=59), respectively. The cases had no metastasis.

The patients had no treatments for bladder cancer before surgery. Patients with Ta and T1 non-muscle invasive bladder cancer were not treated after transurethral resection of bladder tumor (TUR-Bt) until recurrence. When patients with muscle-invasive bladder cancer had recurrence or metastasis after radical cystectomy, they were treated with cisplatin-based chemotherapy.

Blood samples were also collected from 36 healthy donors without malignancy based on the medical history. This study was performed after approval by a local Human Investigations Committee. Informed consent was obtained from each normal and cancer patient.

The sera were separated by centrifugation of the blood after collection, and stored frozen at −80°C until future use for an enzyme-linked immunosorbent assay (ELISA).

### ELISA for Smac/DIABLO

The levels of Smac/DIABLO in the sera were quantitated by the sandwich ELISA according to the manufacturer’s protocol (Assay Designs Inc., Ann Arbor, MI, USA). The serum concentrations of Smac/DIABLO were calibrated from a dose response curve based on reference standards. This method made it possible to estimate serum Smac/DIABLO levels >7.81 pg/ml. Repeated measurements yielded the same results.

Patients were divided into two groups according to the level of Smac/DIABLO in the serum. The level of serum Smac/DIABLO greater than the mean value was regarded as ‘high level’ and the level less than the mean value was regarded as ‘low level’.

### Statistical analysis

All determinations were made in triplicate. For statistical analysis, Student’s t-test was used. Postoperative tumor-free interval and postoperative disease-specific survival rate was determined by the Kaplan-Meier method. The Cox-Mantel test was used to establish the statistical difference in tumor-free period and disease-specific survival rate between the patients with high and low serum Smac/DIABLO levels. P<0.05 was considered significant.

## Results

### Circulating Smac/DIABLO levels in the sera of normal individuals and patients with bladder cancer

**Figure 1:**
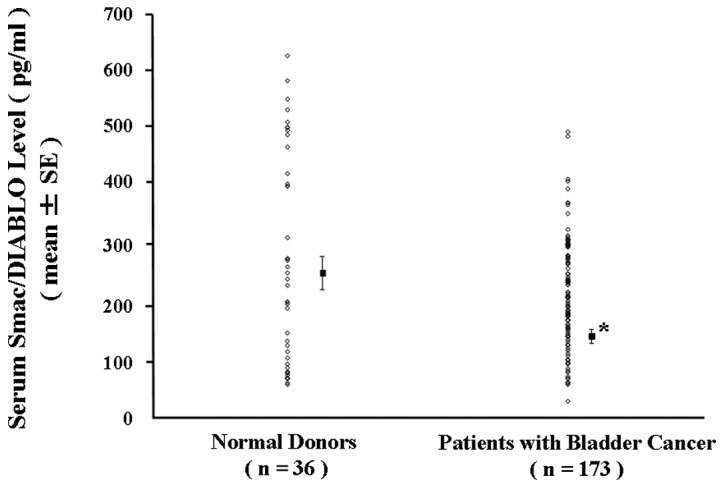
The mean serum level of Smac/DIABLO in patients with bladder cancer and normal donors. The mean serum level of Smac/DIABLO in patients with bladder cancer and normal donors was quantitated by ELISA, as described in Materials and methods. ^*^P<0.05 vs. Normal donors.

The serum levels of Smac/DIABLO in samples derived from normal healthy controls and patients with all histological stages of bladder cancer were determined by ELISA. The mean serum levels of Smac/DIABLO in normal individuals and patients were 257 and 136 pg/ml, respectively (Fig. 1). In comparison with the serum level of healthy individuals, there was an approximately 2-fold decrease in serum Smac/DIABLO level in patients with bladder cancer.

### The serum level of Smac/DIABLO in patients with bladder cancer

**Figure 2:**
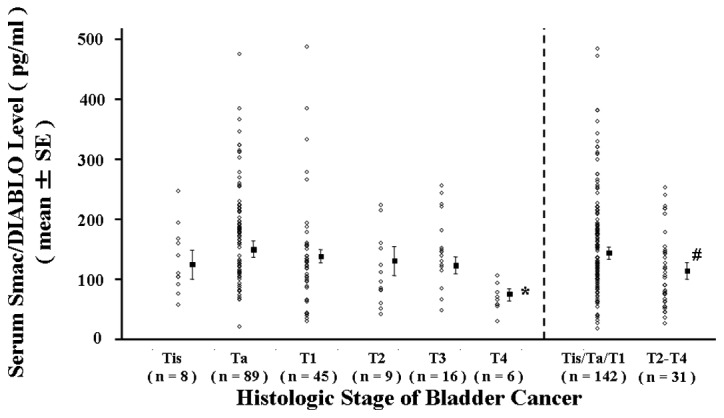
The mean serum level of Smac/DIABLO as a function of the histological stage of bladder cancer. The mean serum level Smac/DIABLO in patients with bladder cancer was quantitated by ELISA as described in Materials and methods. ^*^P<0.05 vs. Ta, T1, T2, T3; ^#^P<0.05 vs. Tis + Ta + T1.

**Figure 3:**
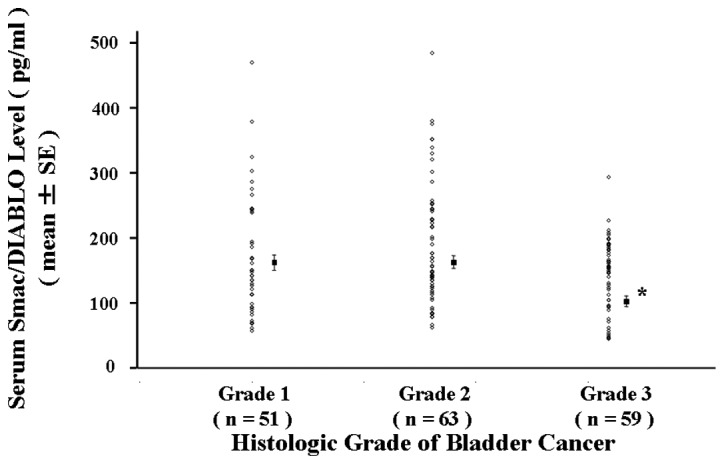
The mean serum level of Smac/DIABLO as a function of the histologic grade of bladder cancer. The mean serum level of Smac/DIABLO in patients with bladder cancer was quantitated by ELISA as described in Materials and methods. ^*^P<0.05 vs. Grade 1, Grade 2.

We examined the serum levels of Smac/DIABLO in patients with bladder cancer as a function of their histologic stages and grades of the disease. The mean serum level of Smac/DIABLO was significantly lower (P<0.05) in patients with muscle-invasive bladder cancer (T2–T4N0M0) than that in patients with non-muscle invasive cancer (Tis, Ta, T1N0M0) (Fig. 2). Furthermore, the mean serum level of Smac/DIABLO in patients with T4 bladder cancer was significantly lower (P<0.05) than that in patients with T2–T3 cancer. The mean serum level of Smac/DIABLO in patients with Grade 3 bladder cancer was significantly lower (P<0.05) than that in patients with Grade 1 and Grade 2 cancers (Fig. 3).

These findings demonstrate that analyses based on the histological stage and grade of bladder cancer revealed a tendency for decrease in the serum level of Smac/DIABLO as a function of disease progression and a higher grade.

### Relationship between the mean serum level of Smac/DIABLO and the postoperative tumor-free period in patients with Ta and T1 non-muscle invasive bladder cancer

**Figure 4:**
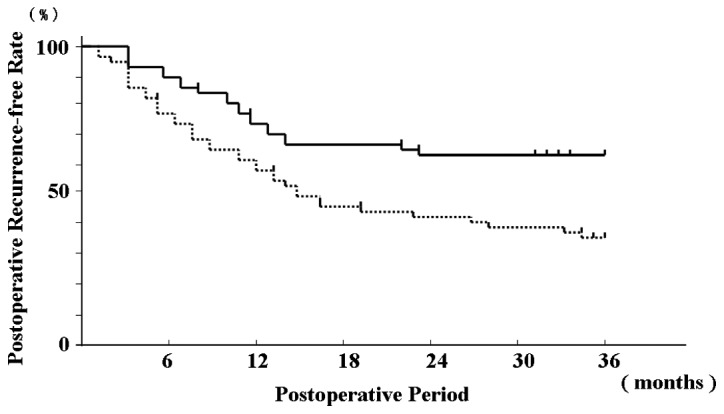
The relationship between the mean serum level of Smac/DIABLO and postoperative recurrence-free rate in patients with Ta and T1 non-muscle invasive bladder cancer. The postoperative tumor-free period of Ta and T1 non-muscle invasive bladder cancer patients undergoing TUR-Bt was determined by the Kaplan-Meier method. Serum levels of Smac/DIABLO greater than the mean value were regarded as high level and serum levels of Smac/DIABLO less than the mean value were regarded as low level. There was a significant difference in tumor-free interval between the following two groups in the 3-year follow-up (P<0.05 by the Cox-Mantel test). **------**, 34 patients with high level of serum Smac/DIABLO; ---, 72 patients with low level of serum Smac/DIABLO.

Ta and T1 non-muscle invasive bladder cancer patients undergoing TUR-Bt were retrospectively evaluated for the postoperative clinical course. The postoperative tumor-free period was estimated by Kaplan-Meier analysis. Based on the analysis, patients with non-muscle invasive bladder cancer were divided into two groups, namely, those with high level of serum Smac/DIABLO (greater than the mean value) and those with low level of serum Smac/DIABLO (less than the mean value). Patients with Ta and T1 bladder cancer with high level of serum Smac/DIABLO showed a significantly longer postoperative tumor-free period as compared to those with low level in the 3-year follow-up (P<0.05) (Fig. 4). These results suggest that the level of serum Smac/DIABLO may be a significant prognostic parameter in patients with Ta and T1 bladder cancer.

### Relationship between the mean serum level of Smac/DIABLO and the postoperative clinical course in patients with T2–T4 muscle-invasive bladder cancer

**Figure 5:**
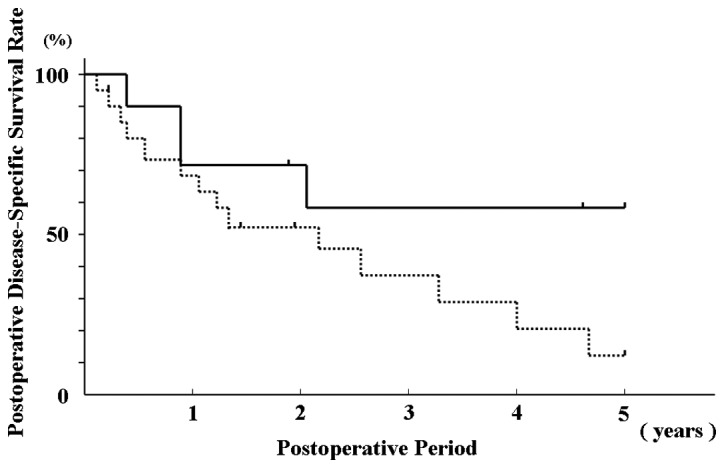
Relationship between the mean level of serum Smac/DIABLO and disease-specific survival rate in patients with muscle-invasive bladder cancer. Postoperative clinical course of T2–T4 (N0M0) muscle-invasive bladder cancer patients undergoing radical cystectomy was determined by the Kaplan-Meier method. Serum Smac/DIABLO levels greater than the mean value were regarded as high level and serum Smac/DIABLO levels less than the mean value were regarded as low level. There was a significant difference in disease-specific survival rate between the following two groups in the 5-year follow-up (P<0.05 by Cox-Mantel test). **------**, 10 patients with high level of serum Smac/DIABLO; ---, 19 patients with low level of serum Smac/DIABLO.

T2–T4 (N0M0) muscle-invasive bladder cancer patients undergoing radical cystectomy were also retrospectively evaluated for the postoperative clinical course. Based on the analysis, patients with muscle-invasive bladder cancer were divided into two groups, namely, those with high level of serum Smac/DIABLO (greater than the mean value) and those with low level of serum Smac/DIABLO (less than the mean value). Patients with muscle-invasive bladder cancer with high level of serum Smac/DIABLO showed a higher disease-specific survival rate as compared to those with low level in the 5-year follow-up (P<0.05) (Fig. 5). These results suggest that the level of Smac/DIABLO may be a significant prognostic indicator in patients with muscle-invasive bladder cancer as well as non-muscle invasive bladder cancer; and that high level of serum Smac/DIABLO may be considered a good prognostic sign.

## Discussion

The present study demonstrates for the first time that Smac/DIABLO can be detected in the serum. Further, we investigated whether the serum level of Smac/DIABLO in patients with bladder cancer was modified as a function of both the stage and the grade of the disease. We report that the mean level of serum Smac/DIABLO in patients with bladder cancer was significantly lower than that in normal volunteers, and that the mean serum level of Smac/DIABLO inversely correlated both with the progression of the stage and the increase of the grade of bladder cancer. To our knowledge, this study is the first to show that patients with Ta and T1 non-muscle invasive bladder cancer patients with high serum level of Smac/DIABLO had a longer tumor-free interval than those with low level in the 3-year follow-up. In addition, patients with muscle-invasive bladder cancer with high serum level of Smac/DIABLO had a higher disease-specific survival rate than those with low level in the 5-year follow-up. Although we report a small number of patients during a short-term follow-up in this study, our preliminary data indicate that the level of serum Smac/DIABLO may be one of the significant prognostic parameters in patients with bladder cancer.

There are many reported studies on the expression of Smac/DIABLO in both normal and cancer tissues (8,9). However, there have been no reports on the serum levels of Smac/DIABLO in either normal donors or cancer patients. Our previous study, using Western blot analysis, revealed that ~24% of patients with bladder cancer had no detectable Smac/DIABLO expression, although all normal bladder specimens expressed Smac/DIABLO. In addition, advanced high grade diseases had lower expression of Smac/DIABLO than those with low stage/grade diseases. Further, our previous study and those of others showed that normal kidney tissues adjacent to the cancer showed higher levels of Smac/DIABLO expression when compared to cancer tissues, and that the progression of renal cell carcinoma was associated with low level of Smac/DIABLO expression (8,10). Similar results were obtained in other cancers including colorectal cancer and lung cancer (11,12). Overall, our studies and those of others demonstrate that the expression level of Smac/DIABLO in cancer tissues is significantly lower compared with the corresponding non-cancerous tissues and correlate with cancer progression. Thus, we have stipulated that serum levels of Smac/DIABLO might have been reflective of the levels of Smac/DIABLO expression in cancer tissues.

The current study demonstrates for the first time that the serum level of Smac/DIABLO predicted the clinical outcome and that the high level in the serum was a good prognostic sign in patients with bladder cancer. The precise underlying mechanism responsible for this relationship remains unclear. Since Smac/DIABLO is a proapoptotic regulatory molecule, it is reasonable to assume that the elevated levels of Smac/DIABLO in the circulation might have resulted from its release following apoptosis of cells. In contrast, the low serum levels of Smac/DIABLO in the serum may reflect tumor escape from apoptosis.

The precise cellular origin of Smac/DIABLO has not been elucidated. We speculate that Smac/DIABLO may be derived from cancer cells and/or normal tissues. Previous studies demonstrated that Smac/DIABLO was expressed by various cancers (8–12). Normal tissues including lung, heart, liver, spleen, pancreas, kidney, prostate, testis and ovary also highly express Smac/DIABLO (4,5). Preliminary experiments showed that Smac/DIABLO was detected in the culture supernatants of bladder cancer cell lines and primary cultures derived from surgical specimens examined. These findings suggest that Smac/DIABLO may be produced by both cancer cells and normal tissues. In addition, bladder cancer cells may secrete several factors that regulate Smac/DIABLO production. Further studies are needed to determine the origin of Smac/DIABLO.

Smac/DIABLO is not a secretory molecule. It is possible that its presence in the circulation may be due to the physiological cell death of normal tissues and cell death of cancer cells. Based on our findings, the circulating levels of Smac/DIABLO in bladder cancer patients on the average are lower than those detected in normal healthy individuals presumably due to a disturbance in the turnover rate of Smac/DIABLO in cancer patients. However, our findings in normal individuals revealed that there was a significant subpopulation of individuals who had levels of Smac/DIABLO in the same range as bladder cancer patients. This may be interpreted that such individuals might have a distinct turnover rate and/or possibly that Smac/DIABLO is degraded and modified and not detected by the ELISA assay used.

Preliminary experiments demonstrate that the level of Smac/DIABLO in the serum derived prior to surgery increased after curative surgery in patients with muscle-invasive bladder cancer (data not shown). However, in patients with non-muscle invasive bladder cancer, the mean serum level of Smac/DIABLO derived before surgery did not change after TUR-Bt. Since Smac/DIABLO is a proapoptotic regulatory molecule, it is reasonable to assume that elevated level of Smac/DIABLO in the circulation may contribute to induce apoptosis in muscle-invasive bladder cancer through its ability to inhibit IAP family members. Therefore, low level of Smac/DIABLO in the serum may be a novel mechanism of tumor cells’ escape from apoptosis in muscle-invasive bladder cancer. If these were the case, the present findings suggest that Smac/DIABLO mimetics may provide a therapeutic means of preventing the growth of muscle-invasive bladder cancer.

IAPs such as XIAP are highly expressed in various cancers and are associated with poor prognosis and resistance to apoptosis (13,14). Our previous report demonstrated that elevated expression of XIAP was found in high stage and high grade bladder cancers (15). Since XIAP blocks apoptosis at the effector phase, strategies targeting XIAP as well as Smac/DIABLO may be effective to overcome resistance to apoptosis. Smac/DIABLO is able to bind to IAP family members and XIAP is predominantly a Smac/DIABLO-binding protein. Smac/DIABLO binds to XIAP, displaces XIAP from caspase-9, promotes cleavage of effector caspases and induces apoptosis (16,17). In addition, Smac/DIABLO is also regulated by IAP family members. IAP family members including XIAP induce the ubiquitination and degradation of Smac/DIABLO (18,19).

Our findings correlating the levels of Smac/DIABLO decreasing as a function of stage and grade of disease might be interpreted by at least two possibilities. The first is that the more aggressive the tumor, the less apoptotic cell death takes place and less Smac/DIABLO is released in the circulation. Alternatively, we have reported that the level of XIAP varies as a function of disease progression, since XIAP induces ubiquitation and degradation of Smac/DIABLO, the more XIAP level in the cell the less Smac/DIABLO will be available and detected in the serum. Furthermore, Smac 3, a Smac/DIABLO isoform, was recently identified (20). Smac 3 induces autoubiquitination and degradation of XIAP. Thus, its low ratio between IAP family members and Smac/DIABLO may be important in favor of the response to cytotoxic therapy. Further studies are therefore needed to determine the regulatory effects of Smac/DIABLO production in bladder cancers. The measurement of IAP family as well as Smac/DIABLO in the serum may be necessary for the accurate evaluation of the prognostic value of Smac/DIABLO and the efficacy of therapy with Smac/DIABLO mimetics.

In conclusion, the data presented in this communication have demonstrated that Smac/DIABLO can be detected in the sera of both normal and cancer patients. The levels of Smac/DIABLO in patients with bladder cancer inversely correlated with histologic stage and grade of the disease; and low serum levels of Smac/DIABLO were associated with early recurrence in patients with bladder cancer. The correlation between the level of serum Smac/DIABLO and postoperative prognosis suggests that the serum level Smac/DIABLO could be used as a prognostic marker in patients with bladder cancer. The accurate prediction of prognosis may help select patients for more intensive surgical or chemotherapeutic approaches in combination with Smac/DIABLO agonists.

## Abbreviations

ELISA: enzyme-linked immunosorbent assay
IAP: inhibitor of apoptosis protein
IBM: IAP-binding motif
MLS: mitochondrial leader sequence
Smac/DIABLO: Second mitochondria-derived activator of caspase/Direct inhibitor of apoptosis protein-binding protein with low pl
TUR-Bt: transurethral resection of bladder tumor
XIAP: X-linked inhibitor of apoptosis protein
